# PYY plays a key role in the resolution of diabetes following bariatric surgery in humans

**DOI:** 10.1016/j.ebiom.2018.12.040

**Published:** 2019-01-11

**Authors:** Claudia Guida, Sam D. Stephen, Michael Watson, Niall Dempster, Pierre Larraufie, Thomas Marjot, Tamsin Cargill, Lisa Rickers, Michael Pavlides, Jeremy Tomlinson, Jeremy F.L. Cobbold, Chun-Mei Zhao, Duan Chen, Fiona Gribble, Frank Reimann, Richard Gillies, Bruno Sgromo, Patrik Rorsman, John D. Ryan, Reshma D. Ramracheya

**Affiliations:** a Oxford Centre for Diabetes, Endocrinology and Metabolism, Churchill Hospital, University of Oxford, Oxford, UK; b Metabolic Research Laboratories and MRC Metabolic Diseases Unit, Wellcome Trust MRC Institute of Metabolic Science, University of Cambridge, Cambridge, UK; c Department of Clinical and Molecular Medicine, Norwegian University of Science and Technology, 7491 Trondheim, Norway; d Translational Gastroenterology Unit, University of Oxford, Oxford, UK; e Oxford Bariatric Service, Oxford University Hospitals NHS Foundation Trust, Oxford, UK; f Oxford Centre for Clinical Magnetic Resonance Research, University of Oxford, UK; g Oxford NIHR Biomedical Research Centre, Oxford, UK

**Keywords:** Bariatric surgery, Diabetes, PYY, IL-22, Gut hormones, Pancreatic hormone secretion, T2D, type 2 diabetes, RYGB, Roux-En-Y gastric bypass, GK, Goto-Kakizaki, BMI, body mass index, PYY, peptide tyrosine tyrosine, GLP-1, glucagon like peptide-1, GSIS, glucose stimulated insulin secretion

## Abstract

**Background:**

Bariatric surgery leads to early and long-lasting remission of type 2 diabetes (T2D). However, the mechanisms behind this phenomenon remain unclear. Among several factors, gut hormones are thought to be crucial mediators of this effect. Unlike GLP-1, the role of the hormone peptide tyrosine tyrosine (PYY) in bariatric surgery in humans has been limited to appetite regulation and its impact on pancreatic islet secretory function and glucose metabolism remains under-studied.

**Methods:**

Changes in PYY concentrations were examined in obese patients after bariatric surgery and compared to healthy controls. Human pancreatic islet function was tested upon treatment with sera from patients before and after the surgery, in presence or absence of PYY. Alterations in intra-islet PYY release and insulin secretion were analysed after stimulation with short chain fatty acids (SCFAs), bile acids and the cytokine IL-22.

**Findings:**

We demonstrate that PYY is a key effector of the early recovery of impaired glucose-mediated insulin and glucagon secretion in bariatric surgery. We establish that the short chain fatty acid propionate and bile acids, which are elevated after surgery, can trigger PYY release not only from enteroendocrine cells but also from human pancreatic islets. In addition, we identify IL-22 as a new factor which is modulated by bariatric surgery in humans and which directly regulates PYY expression and release.

**Interpretation:**

This study shows that some major metabolic benefits of bariatric surgery can be emulated ex vivo. Our findings are expected to have a direct impact on the development of new non-surgical therapy for T2D correction.

**Research in context**

**Evidence before this study**

Bariatric surgery are weight loss interventions which result in rapid and durable reversal of type 2 diabetes (T2D) in man. Changes in gut hormones play a critical role in surgery-induced metabolic improvements.

The gut hormone, GLP-1 has been so far considered as the ‘holy grail’ of this phenomenon although a number of studies have reported that the beneficial effect of the surgery on glucose homeostasis may not depend on GLP-1, thus challenging its unique role.

Like GLP-1, the release of the gut hormone PYY increases after bariatric surgery. A common consensus posits that the main effect of this elevation in humans is on appetite regulation and weight loss post operatively. However, latest evidence from studies done in rodents has shown that, PYY plays a major role in glucose homeostasis and restoration of impaired islet function in severely diabetic rats following Roux-En-Y gastric bypass (RYGB). To date, the relationship between elevated circulating PYY and the metabolic benefits of bariatric surgery on islet secretory function has not been established in humans. Moreover, the impact of factors altered by the surgery on PYY elevation is not been fully characterized in man.

**Added value of this study**

Here we have examined the changes in PYY concentrations six months after bariatric surgery in obese patients compared with healthy control subjects, and their effects on human islet functions. Using a translational approach that combines blood samples from patients with T2D before and after surgery and isolated donor human pancreatic islets, we demonstrate a significant effect of PYY on the improvement of islet secretory function post-surgery. We establish that the short chain fatty acid propionate and bile acids, which are elevated after surgery, can trigger PYY release not only from enteroendocrine cells but also from human pancreatic islets. In addition, we identify IL-22 as a new factor which is modulated by bariatric surgery and which may directly regulate PYY expression and release, elucidating some of the mechanisms behind increased PYY concentrations after bariatric surgery in humans.

**Implications of all the available evidence**

This study demonstrates that the metabolic benefits of bariatric surgery on islet secretory functions can be emulated ex vivo, giving rise to the possibility of identifying the critical mediators of these effects. Our findings that the gut hormone PYY mediates some key anti-diabetic effects of bariatric surgery imply that a pharmacological agent enhancing PYY release or its action could provide an effective and non-surgical therapy for T2D. Moreover, the identification of factors specifically triggering PYY induction both systemically and within the islets, may pave the way for an alternative cure to peptide therapeutics, thus overcoming their inherent weaknesses, like poor pharmacokinetic properties and stabilities, and yet achieving the same benefits for the treatment of diabetes.

Alt-text: Unlabelled Box

## 1 Introduction

Bariatric surgery results in rapid and durable reversal of type 2 diabetes (T2D) and associated metabolic benefits in man [1]. Changes in gut hormones play a critical role in surgery-induced metabolic improvements, which are independent of weight loss [2]. In particular, post-operative elevation in the incretin hormone GLP-1, has been reported to contribute to appetite suppression and improved glycaemic control [2]. However, recent studies done in rodents and humans have revealed the existence of GLP-1-independent mechanisms, indicating that multiple cofactors mediate the metabolic effects of surgery [[3], [4], [5], [6], [7]]. Moreover, increased levels of another intestinal hormone called peptide tyrosine tyrosine (PYY) have been reported following bariatric surgery. This peptide hormone was found to be central in the reversal of hyperglycaemia and restoration of impaired insulin release following Roux-en-Y gastric bypass (RYGB) in a rat model of T2D [8].

Both fasting and postprandial PYY levels increase significantly within just one-week of RYGB or sleeve gastrectomy (SG) in obese patients, and remain elevated after 1 year [[9], [10], [11]]. These changes are associated with normalization of fasting glucose levels, and improved blood pressure, hypercholesterolemia and hypertriglyceridemia [12]. Chronic treatment of human isolated islets from diabetic and non-diabetic donors with exogenous PYY enhances glucose-mediated insulin secretion and improves suppression of glucagon, suggesting a direct effect of PYY on the correction of human diabetes [8,13]. However, the relationship between elevated circulating PYY and the metabolic benefits of bariatric surgery on islet secretory function and diabetes reversal has not been explored in humans.

PYY is mainly secreted from the L-cells of the ileum and colon in response to food intake and, to a lesser extent, it is also expressed in the delta-, PP- and alpha-cells of the pancreatic islets [13]. In clear contrast to healthy islets, PYY content has recently been shown to be reduced by 50% in islets from the Goto-Kakizaki (GK) rat model of T2D, suggesting a role of islet-derived PYY in health and diabetes [13]. Moreover, consistent with the changes in circulating levels, PYY content in islets is markedly increased following RYGB in diabetic rats [13], potentially contributing ing to RYGB-induced restoration of impaired islet morphology and function. Several factors including accelerated nutrient delivery to the intestine are likely to stimulate PYY secretion following weight-loss surgery. Emerging evidence also suggests that alteration in bile acids, and the intestinal microbiota and their metabolic products can induce PYY release from enteroendocrine cells [[14], [15], [16], [17]]. However, whether they can also modulate secretion of PYY from pancreatic islets, especially in humans is not known. One of the well-described effects of bariatric surgery is a reduction in the chronic systemic inflammation associated with obesity. Indeed, levels of inflammatory cytokines, such as IL-6 and TNFα decrease after bariatric surgery [[18], [19], [20]]. Unlike other cytokines, interleukin-22 (IL-22), a small cytokine mainly produced by Th1, Th17, Th22 and ILC3 cells, has been shown to alleviate metabolic disorders in mice and protect pancreatic islet properties beyond its immunological functions [21,22]. Importantly, infusion of IL-22 in diet-induced obese mice has been shown to result in elevated circulating PYY levels, which in turn reduces food consumption. To date, the role of IL-22 in weight-loss surgery and diabetes remission in man has not been explored, and its potential impact on islet-derived PYY has not been determined.

Here we have examined the changes in PYY concentrations six months after bariatric surgery in obese patients compared with healthy control subjects, and whether they can influence human islet secretory functions. In addition, we have assessed the impact of short chain fatty acids and bile acids on PYY release and glucose-mediated insulin secretion from isolated pancreatic human islets. Finally, we have explored alterations in plasma IL-22 concentrations following bariatric surgery in humans and modulation of PYY release from pancreatic islets by IL-22.

## 2 Materials and methods

### 2.1 Patients

All patients undergoing bariatric surgery (sleeve gastrectomy or RYGB) for morbid obesity have been recruited into the Oxford GI Biobank (*Gastrointestinal Illness in Oxford: prospective cohort for outcomes, treatment, predictors and biobanking with ethical approval from Yorkshire & The Humber - Sheffield Research Ethics Committee, REC Ref:*^11^*/YH/0020 and 16/YH/0247*) *REC Ref:*^11^*/YH/0020 and 16/YH/0247*), since November 2014. Written informed consent was obtained from all study subjects. Blood samples were collected on the day of surgery, and at 6 and 12 months post-operatively, along with detailed clinical information. Pre-operation samples were taken under fasting conditions, while follow up samples were non-fasting, owing to the logistics of starving post-surgical patients, and afternoon clinic visits. However, the timing of blood sampling from the patients is expected to have minimal or negligible effects on islet hormone secretion. Patient characteristics are outlined in Table S1. Healthy control samples were obtained from the Oxford Centre for Clinical Magnetic Resonance Research (OCMR; *Non-Invasive and Comprehensive Liver Assessment (NICOLA) study; REC Ref*^13^*/SC/0243*), details outlined in Table S1.2.

### 2.2 Animals

Female C57BL6J mice (Charles River, US), aged 16–20 weeks, were used for islet functional studies and female mice bred in house on a C57BL/6 background, aged 16 to 24 weeks were used for colonic primary culture. Adult male 16–20 weeks-old Wistar rats as normoglycaemic controls and age and sex matched diabetic GK rats (Taconic, Denmark) were used as a model of type 2 diabetes. Data from experiments done in mice and rats are not cross compared with data generated from rats. All animal experiments were conducted in accordance with the UK Animals Scientific Procedures Act (1986) under the UK Home Office Project License 70/7824 and were approved by the University of Cambridge Animal Welfare and Ethical Review Body.

### 2.3 Islet isolation

Mouse islets were isolated by liberase (Sigma-Aldrich Ltd., Gillingham, UK) digestion as described previously [23].

Human pancreases were obtained with ethical approval and clinical consent from non-diabetic donors. Islets were isolated in the Diabetes Research & Wellness Foundation Human Islet Isolation Facility by collagenase digestion (Serva) using modified versions of published protocols [24]. The donor isolated human islets that were used for secretion studies were obtained with research consent via the DRWF Human Islet Isolation Facility at which was established in 2006 in the Oxford Centre for Diabetes, Endocrinology, and Metabolism in Oxford. The clinical islet isolation facility is a purpose built, clean-room laboratory, licensed by the Human Tissue Authority for the aseptic production of pancreatic islet cells from cadaveric donor pancreases. It plays a pivotal role in the supply of islets within the UK Islet Transplant Consortium (UKITC) for the delivery of an NHS funded national therapy for the treatment of type 1 diabetes in patients who have unstable diabetes with severe complications. When islets cannot be transplanted and where there is research consent, the isolated islets are made available for high quality diabetes research projects. Human islets were used for experiments as they became available. The robustness of each islet preparation is evaluated by the glucose-mediated insulin secretion index, which is calculated by dividing the mean insulin secretion at 20 mM glucose (stimulatory concentration) by that at 1 mM glucose (basal concentration). Thus, a prep which is not glucose-responsive is regarded as poor quality and one with at least a two-fold elevation in insulin secretion in response to 20 mM glucose, is regarded as good quality islets. Donor details are provided in Table S2.

### 2.4 Roux-en-Y gastric bypass model

Sham or RYGB surgery was performed on anesthetized rats as previously described [8]. Briefly, the intestine was transected 10 cm distal to the ligament of Treitz, creating a distal and a proximal end. The proximal end was anastomosed to the intestine and a gastric pouch was created (2–3% of total stomach). The distal end of the intestine was anastomosed to the gastric pouch in an end-to-side fashion. For sham operation the animals were opened through a midline incision and the viscera were gently manipulated.

### 2.5 Measurements of serum concentrations of PYY, GLP-1 and IL-22

Subject details are reported in Table S1.1 and S1.2. Human serum was kept at −80 °C until determination of hormones. Total PYY and glucagon were measured by radioimmunoassay (Millipore and Eurodiagnostica). Total GLP-1 and insulin were assayed using human triplex kit (K15160C, Mesoscale Discovery). IL-22, IL-6 and IL-23 were measured in plasma and serum samples by Human IL-22 or IL-6 or IL-23 quantitative Elisa Kit (R&D systems).

For GK rats, blood was drawn from abdominal aorta at sacrifice under ad-libitum feeding conditions and processed as described previously [25]. IL-22 was measured in serum samples by means of mouse/rat IL-22 quantitative Elisa Kit (R&D systems).

### 2.6 Hormone secretion studies

Serum samples from the same patients before and 6 months after bariatric surgery were used (Table S1.1). Islets were pre-cultured for 72 h in RPMI (5 mM glucose) with the addition of 20% pooled serum in absence or presence of exendin (9–39; 1 μM; Bachem) or anti-PYY antibody (1:500; ab22663, Abcam) as previously described [8]. For each experiment, sera from two patients were pooled. Secretion experiments were set with 12 hand-picked and size-matched islets per tube (in triplicate). Islets were pre-incubated in Krebs-Ringer buffer (KRB) containing 2 mg/mL BSA and 3 mmol/l glucose for 1 h at 37 °C, followed by 1 h test incubation in KRB supplemented with glucose as indicated. Insulin and glucagon content were determined by radioimmunoassay (Millipore and Eurodiagnostica, respectively) according to the manufacturer's instructions. To allow comparison between experiments, secretion data are presented as mean percent basal, where basal is secretion at 1 mM glucose.

Secretion experiments under acute or chronic conditions were performed as previously reported [13].

### 2.7 Measurements of PYY release from islets

20 islets per condition were cultured in 500 μL medium in absence or presence of propionate, butyrate and acetate (1 mM; Sigma-Aldrich) or GPBAR-A (30 μM; Sigma-Aldrich) or recombinant IL-22 (100 ng/mL; R&D systems), each condition was set in triplicates. After the incubation time, islets and medium were collected separately and stored at −20 °C until determination of hormones. Total PYY was measured in islet and in the medium by radioimmunoassay (Millipore (human) and Phoenix Pharmaceuticals (mouse) according to the manufacturer's instructions. PYY release was normalized over the intra-islet PYY content to minimize any variations due to differences in islet size. Analysis of intra-islet PYY content only was performed in parallel to ensure no specific effect of the treatment on the total PYY levels in islets.

### 2.8 Gene expression analysis in colonic primary culture

Colonic primary cultures were prepared as previously described [26,27] Colon and rectum were quickly isolated from mice sacrificed by cervical dislocation, washed thoroughly with PBS and cleaned of their outer muscle layer. Minced tissue was digested with 0.4 mg/mL Collagenase XI (Sigma-Aldrich) at 37C to isolate crypts. Resuspended crypts were plated on Matrigel coated wells in culture media (DMEM high glucose supplemented with FBS10%, l-glutamine (2 mM), penicillin (100 U/mL), streptomycin (0.1 mg/mL) and Y27632 (10 μM, Tocris)). Plated crypts were washed the day after with fresh media and treated for 72 h accordingly in duplicates, changing the media after the first 24 h. Media was removed and replaced by Trizol (Sigma) and RNA extracted using manufacturer's protocol and DNA removed incubating RNA for 20 min at 37C with DNAse I (Invitrogen). 200 ng of RNA were reverse transcripted (High capacity cDNA Reverse Transcription kit, Thermo Fisher) and qPCR using Taqman probes and Taqman Fast universal Master mix (Life technologies) was performed on an ABIquantStudio 7 (ThermoFisher Scientific) for *Pyy*, *Gcg* and β*-actin* (Mm00520716, Mm00801714 and Mm02619580, Life technologies). Data was analysed using the 2^-∆∆Ct^ method with β-actin as reporter gene and using the mean of the control duplicates as control. Statistical difference was evaluated using a Dunn to control test from three different experiments done in duplicates (R, PMCMR package).

### 2.9 PYY peptide quantification in colonic primary culture

The protein fraction from the Trizol extraction was extracted: after the DNA precipitation, protein are precipitated with 1.5 v/v cold acetone for 10 min, and the protein pellet after a 12,000 *g* 10 min centrifugation at 4C is washed once with 1 mL of 0.3 M guanidine HCl in 95% ethanol, once with 100% EtOH for 20 min followed by a 5 min 7500 *g* centrifugation at 4C. After the last wash, samples are air dried and resuspended in 8 M urea by incubation 30 min at 42C. Proteins are then precipitated with 800uL ACN80% (in water) and peptides collected in the supernatant after 12,000 *g* 4C 10 min centrifugation and dried by vacuum centrifugation. Peptides resupended in 0.1% formic acid are then purified using a solid phase extraction (Oasis prime HLB, Waters) and eluted in 2 × 30 μL 60% MetOH, 30% H2O, 10% acetic acid and diluted with 75 μL 0.1% formic acid in solution.

Peptides were analysed on an H-Class Acquity (Waters) attached to a TQ-XS triple quadrupole mass spectrometer (Waters). Sample (20 μL) was injected onto a 2.1 × 50 mm 1.8 μm particle HSS T3 Acquity column held at 60 °C and flowing at 350 μL/min. Gradient starting condition was 99% A (0.1% formic acid in water v/v) and 1% B (0.1% formic acid in ACN). Starting conditions were held for 0.25 min before raising to 50% B over 7 min. The column was flushed with 90% B for 1 min before returning to starting conditions. The total time of each analysis was 10 min, with the first 0.75 min and last 2 min diverted to waste. Mass spectrometry conditions involved targeting four peptides using the transition and collision energy details given below:

**Table:** 

Peptide	Q1 (*m/z*)	Q3 (*m/z*)	Collision energy (eV)	Dwell time (ms)
PYY 1–36 1	664.4	732.4	20	25
PYY 1–36 2	664.4	754.4	20	25
PYY 3–36 1	707.7	847.5	20	25
PYY 3–36 2	707.7	871.9	20	25
Oxyntomodulin 1	742.6	680.7	22.5	25
Oxyntomodulin 2	742.6	923.7	22.5	25
GLP-II 1	943.0	1306.58	25	25

The source parameters used included a positive electrospray ion voltage of 3.0 kV, gas flow of 1000 L/h, desolvation temperature of 600 °C and a cone voltage of 40 V. Peptide peak areas were integrated using the TargetLynx program associated with Masslynx V 4.2 (Waters).

### 2.10 Statistical analysis

Values are expressed as the mean ± standard error of the mean (SEM). The statistical significance of differences was evaluated by the Unpaired Student's *t*-test assuming unequal variation when two groups were compared or by one way ANOVA and Bonferroni's multiple comparison tests when multiple groups were compared. A p value of <0.05 was regarded as significant. Statistical analysis was performed using GraphPad Prism.

Correlation analysis was performed with Pearson's pairwise correlation test (GraphPad).

## 3 Results

### 3.1 Obese subjects have low circulating PYY levels, which are restored post-bariatric surgery

**Fig. 1:**
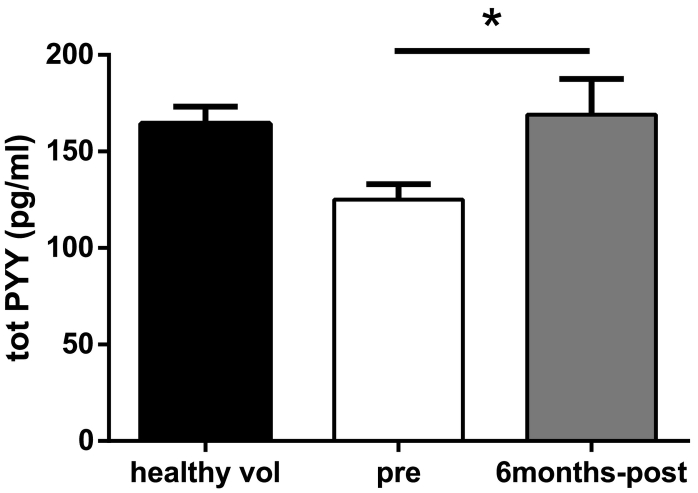
Changes in PYY concentrations in human serum samples. Serum total PYY in healthy volunteers (n = 20) and in patients before and 6 months after bariatric surgery (n = 25). Data are presented as mean ± SEM. (One-way ANOVA for multiple comparison) ^⁎^P < 0.05 for indicated comparison.

Serum total PYY levels were measured in obese patients before (mean ± SEM; BMI 47 ± 8.4) and six months after either gastric bypass or sleeve gastrectomy (SG; mean ± SEM; BMI 37.5 ± 7.4; p < 0.0001) and compared with healthy volunteers (mean ± SEM; BMI 22.24 ± 2.1) (Fig. 1). Serum PYY concentrations were 25% lower in obese individuals compared to healthy people (mean ± SEM in pg/mL; 125 ± 8.1 vs 164.5 ± 8.8, p-value = 0.002). Following both RYGB and SG, serum PYY in obese patients increased to levels seen in normal-weight individuals. Total GLP-1 levels were not significantly changed by bariatric surgery (Fig. S1a). Circulating insulin levels, which were markedly raised in obese individuals, decreased post-operatively (Fig. S1b).

### 3.2 Improvement of human islet function post-surgery is mediated by PYY

**Fig. 2:**
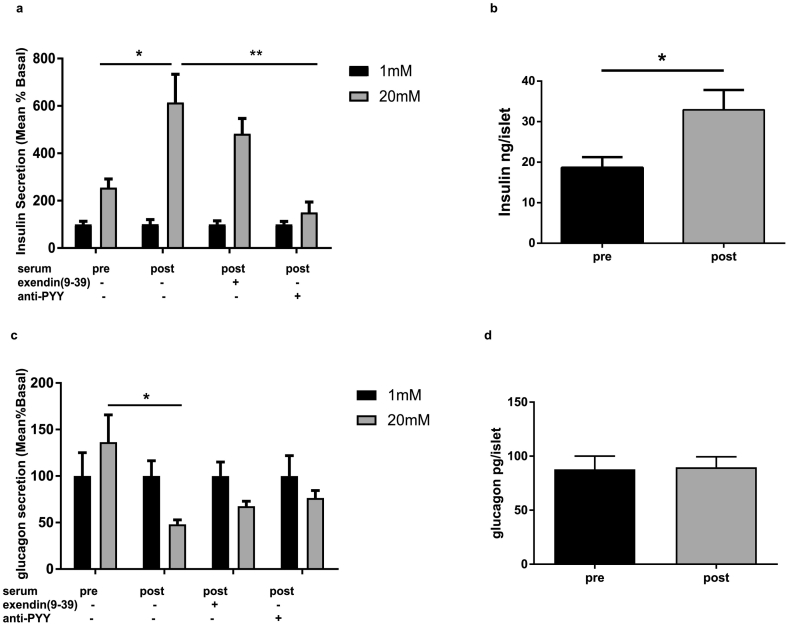
PYY mediates metabolic benefits of bariatric surgery. Insulin (a) and glucagon (c) secretion from human islets (n = 3 donors n = 3) treated for 72 h with serum from patients before (pre) and 6 months after surgery (post) in absence or presence of exendin [[9], [10], [11], [12], [13], [14], [15], [16], [17], [18], [19], [20], [21], [22], [23], [24], [25], [26], [27], [28], [29], [30], [31], [32], [33], [34], [35], [36], [37], [38], [39]] or PYY antibody. Secretion was measured in islets stimulated with 1 mM (black bars) or 20 mM glucose (grey bars). Data are presented as percentage of basal secretion (mean ± SEM as percentage of content). Insulin (b) and glucagon (d) content in human islets treated for 72 h with serum from patients before (pre) and 6 months after surgery (post). (One-way ANOVA for multiple comparison) ^⁎^P < 0.05, ^⁎⁎^P < 0.01for indicated comparison.

Isolated human islets from three donors were cultured for 72 h in the absence and presence of serum collected from patients before and after bariatric surgery Following the culture period, insulin and glucagon release were measuredat low (1 mM) and high (20 mM) glucose concentrations. Islets exposed to pre-surgery serum exhibited a lack of insulin release in response to 20 mM glucose (Fig. 2a). In contrast, application of the serum collected post-surgery resulted in a three-fold increase in glucose-mediated insulin secretion, with no significant alteration in the basal insulin level (Fig. 2a). Islets treated with the post-surgery serum also exhibited normal suppression of glucagon at 20 mM glucose (Fig. 2c). We hypothesized that humoral factors altered by surgery, including circulating gut hormones, mediate the improvements seen in insulin and glucagon secretion from islets. To further test the possible involvement of systemic GLP-1, the GLP-1 receptor antagonist exendin [[9], [10], [11], [12], [13], [14], [15], [16], [17], [18], [19], [20], [21], [22], [23], [24], [25], [26], [27], [28], [29], [30], [31], [32], [33], [34], [35], [36], [37], [38], [39]] was added to the serum during culture. Under these conditions, GLP-1 receptor blockade did not affect glucose-mediated insulin secretion. To explore if PYY is the humoral factor mediating the beneficial effects of bariatric surgery on islet function in man, islets from human donors were co-incubated with the post-surgery serum and a PYY-specific antibody at a concentration demonstrated to react with the peptide [13]. Immuno-neutralization of PYY resulted in complete reversal of glucose-induced insulin release (Fig. 2a). Chronic treatment of human islets with post-surgery serum also resulted in increased insulin content (Fig. 2b), in agreement with the effects of exogenously applied PYY on isolated islets [13]. On the other hand, both inhibition of GLP-1 signaling and immunoneutralization of PYY showed a tendency to partially attenuate the effects of post-surgery serum on glucagon release (Fig. 2c). There was no change in islet glucagon content (Fig. 2d).

### 3.3 Which factors, altered by bariatric surgery, cause PYY elevation?

#### 3.3.1 Propionate and bile acids mimic the effects of bariatric surgery by inducing pancreatic PYY release and potentiating insulin secretion

**Fig. 3:**
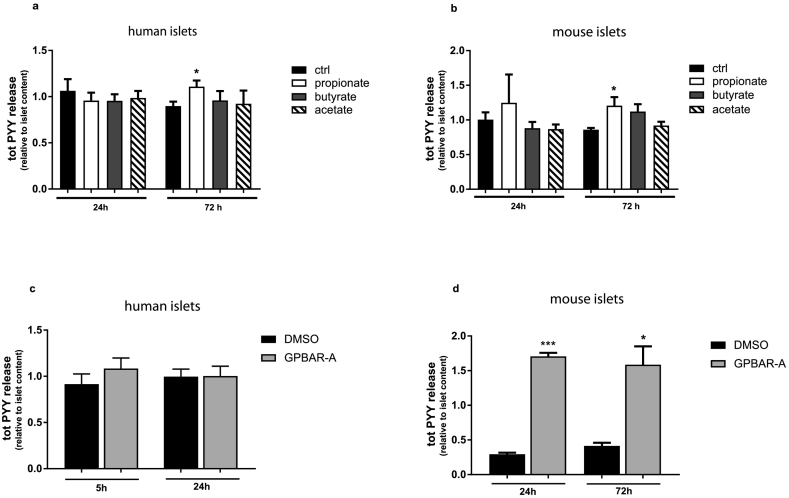
Propionate and bile acids induce pancreatic PYY release. Human (donors n = 9) (a, c) and mouse (mice n = 3) (b, d) islets were incubated with 1 mM propionate, butyrate or acetate or 30 μM GPBAR-A for the indicated time length (included in culture medium). Ratio between total PYY in the medium and in the islets is reported. ^⁎⁎⁎^P < 0.001, ^⁎^P < 0.05 for indicated comparison.

Bacterial-derived metabolites, such as short chain fatty acids increase in rodents and man following bariatric surgery [29,30] and can directly modulate PYY secretion from L-cells [31,32]. To test if SCFAs can also influence PYY release from islets ex vivo, mouse and human islets were treated with propionate, butyrate and acetate. Of the three SCFAs, only propionate resulted in a small but significant increase in PYY secretion at 72 h in both human and mouse islets (Fig. 3a and b). No difference in islet PYY content was observed between control and propionate treatment at any time point (data not shown). Chronic incubation of mouse islets with propionate also potentiated glucose-stimulated insulin secretion (Fig. S2a), in agreement with previous findings [33].

Alteration in the enterohepatic circulation of bile acids is thought to be another important mediator of the weight-independent benefits of bariatric surgery [34]. Like SCFAs, bile acids can induce GLP-1 and PYY release from enteroendocrine cells via activation of the bile acid receptor TGR5 [35] which is also expressed on pancreatic islets [36]. Incubation of human islets with GPBAR-A, a potent agonist of TGR5, had no effect on islet-derived PYY release (Fig. 3c) but resulted in enhancement of glucose-stimulated insulin secretion (Fig. S2b), recapitulating previous findings in rodents [36]. In contrast to human islets, GPBAR-A strongly elevated PYY secretion in mouse islets, and this effect was sustained even after prolonged incubation (Fig. 3d).

#### 3.3.2 IL-22 is a new factor induced by bariatric surgery

**Fig. 4:**
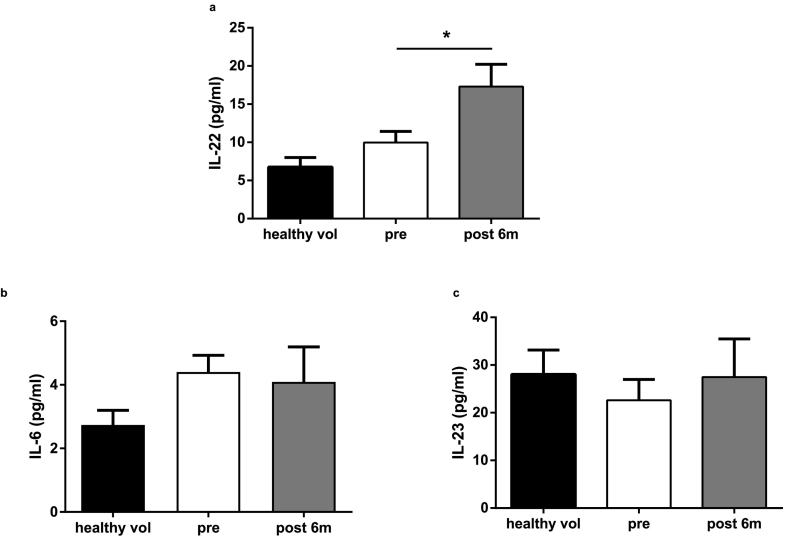
IL-22 levels increase after bariatric surgery. Plasma IL-22 (a), IL-6 (b) and IL-23 (c) in healthy volunteers (n = 20) and in patients before and 6 months after bariatric surgery (n = 25). Data are presented as mean ± SEM. (One-way ANOVA for multiple comparison) ^⁎^P < 0.05 for indicated comparison.

A number of circulating inflammatory cytokines known to be elevated in obesity are reduced following bariatric surgery, possibly as a consequence of the substantial weight loss that occurs. IL-22 is an inflammatory mediator of the innate and adaptive immune response which also regulates adiposity and metabolic disorders [37]. Circulating IL-22 levels were assessed in healthy volunteers and obese patients before and six months after bariatric surgery. Compared to levels before surgery, a significant increase in IL-22 concentrations was observed in sera from post-surgery patients (Fig. 4a). A trend towards increased IL-22 levels was also found in obese individuals in comparison to healthy people. To test whether such elevation was conserved in rodents after bariatric surgery we also explored IL-22 concentrations in serum samples from lean, diabetic GK rats after RYGB or sham surgery. Compared to levels in the sham control sera, a more pronounced elevation in IL-22 concentrations was noted in sera from GK rats post-RYGB (Fig. S3a). In contrast to IL-22, circulating levels of IL-6 and IL-23 were not altered by the surgery in humans (Fig. 4b and c).

#### 3.3.3 IL-22 modulates PYY expression and release from enteroendocrine cells and pancreatic islets

**Fig. 5:**
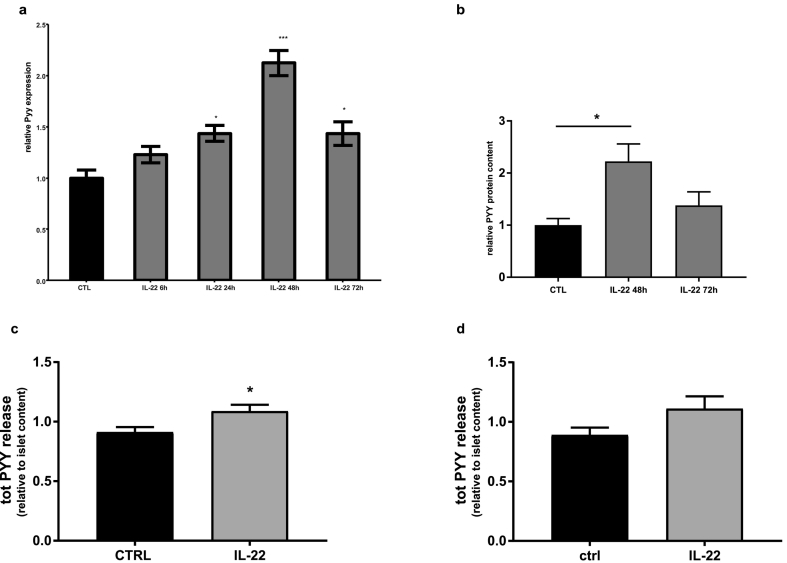
IL-22 induces PYY gene expression and PYY secretion in pancreatic islets and primary colonic culture. (a, b) Expression of PYY gene (a) and protein (b) in colonic primary mouse culture non-treated (NT) or exposed to 100 ng/mL IL-22 for 6, 24, 48 and 72 h (mice n = 4). Data are presented as relative *Pyy* expression/protein to control (mean ± SEM). Human (b) and mouse (c) islets were treated with 100 ng/mL IL-22 for 24 h. Ratio between total PYY in the medium and in the islets is reported. ^⁎^P < 0.05 for indicated comparison.

In our analysis the increase in circulating PYY after surgery correlates with the increase in circulating IL-22 levels (Pearson r: 0.73) (Fig. S3b). To test whether IL-22 directly induces PYY expression from enteroendocrine cells, mouse colonic primary cultures were treated with IL-22 for 6, 24, 48 and 72 h and *Pyy* mRNA levels were measured. Stimulation with IL-22 for 24 to 72 h significantly increased *Pyy* gene expression with the highest peak at 48 h incubation (Fig. 5a). Such elevation was further confirmed at protein levels in the colonic culture extracts by LC-MS (Fig. 5b).

In parallel, the effects of IL-22 on PYY release from pancreatic islets were studied. Twenty-four hours exposure of isolated mouse and human islets with the recombinant cytokine resulted in a small but significant increase in PYY release (Fig. 5c and d).Of note, while the presence of IL-22 receptors on human pancreatic islets has been documented, we were unable to detect IL-22 in isolated islets [38].

IL-22 has been associated with preservation of insulin secretion in isolated islets upon induction of cytokine-mediated oxidative and ER stress. However, under physiological conditions, it does not affect glucose-induced insulin release [22]. In line with this observation, acute (1 h) and chronic (72 h) treatment of healthy mouse and human islets with IL-22 did not affect glucose-induced insulin release (Fig. S4), suggesting that a direct compensatory effect of IL-22 occurs only on compromised beta-cell function.

## 4 Discussion

A combination of factors, rather than a single one, is likely to account for the compelling metabolic benefits associated with bariatric surgery. PYY has been described as a classical weight loss-mediator and its role in the metabolic benefits of surgery has largely been limited to its function as an appetite regulator. Here we show that, compared with levels in healthy volunteers, total PYY levels are significantly lower in obese people before surgery confirming its negative correlation with body mass index (BMI) [39]. Sleeve gastrectomy and gastric bypass are the two most effective surgical procedures for weight loss and the induction of remission in diabetes [40]. In association with a substantial reduction in BMI in all patients six months post-surgery, a clear elevation in circulating total PYY levels was noted after both types of operations. In contrast, there was no significant change in total GLP-1 levels in the same serum samples at six months post-surgery. These findings are in agreement with previous observations documented over the long-term following surgery [5,41,42]. Along with significant weight loss, a reduction in HbA1c was observed in 71% of the patients in keeping with improved glucose regulation. More recently, PYY has been described as a critical factor in the restoration of impaired islet secretory function after gastric bypass in the GK rat model of T2D [8] and exogenous application of recombinant PYY has been demonstrated to potentiate glucose-dependent insulin secretion in human islets from diabetic donors [13]. However, an association between increased circulating PYY concentrations and improved islet function post bariatric surgery in humans has not been investigated previously. Using donor human islets ex vivo and pre- and post-surgery sera, we show that improved glucose-induced insulin and glucagon secretion that occurs post-surgery can be emulated in vitro. In particular, prolonged exposure of islets with sera post-surgery restored the glucose-mediated inhibition of glucagon secretion, which was lacking in islets exposed to pre-surgery sera, whereby glucose tended to stimulate glucagon secretion, an observation consistent with those reported in islets from patients with T2D [28]. These findings demonstrate that bariatric surgery can influence islet function via blood-borne factors, which remain conserved in serum. We also report that immuno-neutralization of PYY leads to complete reversal of surgery-induced improved insulin secretion and a partial, although not significant, reversal in glucagon release from human islets treated with post-surgery serum. These data indicate that in man, PYY acts as the humoral factor which mediates the beneficial effects of bariatric surgery on islets. It is noteworthy that chronic treatment of islets with post-surgery serum induces the same fold increase in insulin content as previously reported following chronic exposure of islets to recombinant PYY [13], implying that elevation of insulin content in islets may be crucial for ameliorating beta-cell secretory properties.

**Fig. 6:**
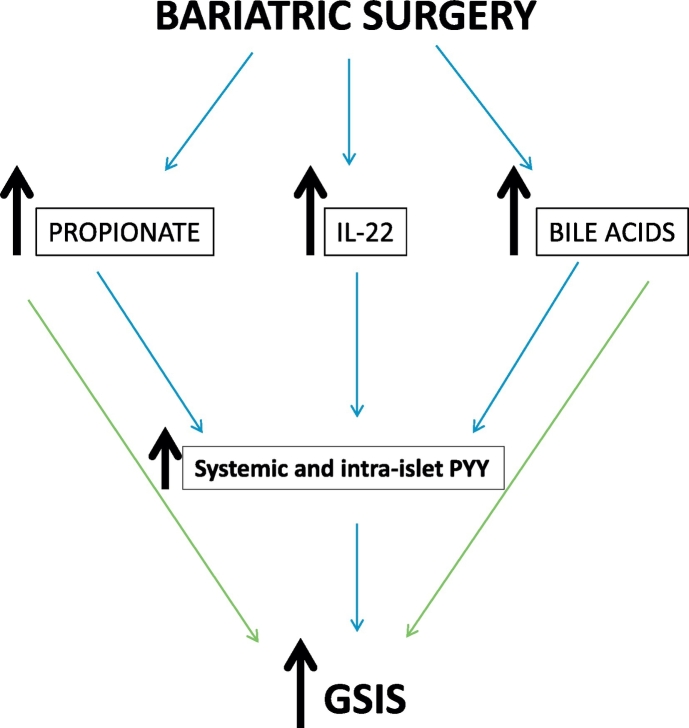
Increased PYY levels in circulation and in islets occur via several factors and jointly contribute to restoration of islet function after bariatric surgery. GSIS = glucose-stimulated insulin secretion.

In addition to elevating circulating PYY levels in the GK rats, gastric bypass surgery also leads to an increase in pancreatic PYY content (compared to sham-operated controls) [13], thus providing a local (intra-islet) source of PYY that may directly affect islet secretory functions. However, the mechanism behind PYY elevation in islets post-surgery remains unknown. Bariatric surgery significantly influences gut microbiota by altering the abundance of specific bacterial *Phyla* [43]. Short chain fatty acids (SCFAs) are products of bacterial fermentation with acetate, propionate and butyrate being the most abundant. Increased levels of SCFAs have been reported in rodents and man following bariatric surgery, in particular after RYGB [29,30]. Like bacterial-derived metabolites, bile acids are altered by bariatric surgery and are known to modulate the release of gut hormones [35,44] and beta-cell function [33,36]. However, their impact on islet-derived PYY has not been studied previously. Our results demonstrate that prolonged treatment with the short chain fatty acid propionate can lead to a modest increase in PYY release from mouse and human isolated islets. Although this delayed response cannot be fully explained, we speculate that it might be secondary to alteration in gene expression and expression of intermediate factors. In addition, the activation of the bile acid receptor TGR5 which is expressed on pancreatic beta-cells [36], results in a prominent induction of islet-derived PYY in mice. This effect was not observed in human islets, suggesting a possible species-specific impact of bile acid changes on gut-dependent PYY production. Bile acids are increasingly recognized as important regulators of lipid and glucose metabolism [45]. In patients, fasting and postprandial bile acid concentrations rise following RYGB, while conflicting results have been reported after SG [34,46]. Restoration of glucose-dependent insulin secretion post-surgery could be attributed to increased levels of bile acids since activation of the bile acid sensors FXR [47] and TGR5 [36] has been shown to enhance insulin release in isolated islets. Consistent with these reports, we observe that 1 h stimulation of TGR5 by the agonist GPBAR-A potentiates glucose-stimulated insulin secretion in human islets, even when the donor human islets were poorly responsive to glucose, probably due to their sub-optimal quality (purity <50%; viability<50%). Collectively these data indicate that propionate and bile acids can enhance glucose-induced insulin secretion both directly and indirectly via increased release of PYY from the gut and islets (Fig. 6).

Obesity and the metabolic syndrome are accompanied by a ‘low grade’ chronic inflammation, indicated by a well-characterized positive association between obesity indices and inflammatory markers, such as C reactive protein (CRP) and IL-6 [48]. Thus, weight loss triggered by bariatric surgeries is expected to lead to a reduction in the circulating levels of inflammatory mediators, often seen more than six months after the operation [18,19]. To date, there is a lack of consensus regarding the changes in IL-6 levels following weight-loss surgery. Whereas, some studies have documented a substantial post-operative decrease [[49], [50], [51]], Miller et al. has reported no significant changes in IL-6 six months after gastric bypass [20]. These latter reports are consistent with our findings in which IL-6 and IL-23 levels were unaffected by bariatric surgery in humans. On the other hand, post-surgery elevation in IL-22 that we observe here is a novel finding deserving further study. In the last years the roles of this cytokine have been extended beyond host defense and inflammation in the intestine and include protective effects in different metabolic conditions. For example, IL-22 influences lipid metabolism in the liver, reduces lipogenesis and ameliorates hepatic steatosis induced by high fat diet [52]. Thus, it is plausible to speculate that its elevation after bariatric surgery might contribute to the improvement in steatosis and nonalcoholic fatty liver disease reported in patients [53].

Unlike other cytokines, IL-22 also protects pancreatic islets from oxidative and ER stress and preserves their secretory functions, whilst opposite effects are mediated by IL-23, a cytokine upstream of IL-22 [22]. Due to its unique role in alleviating metabolic disorders and its direct restorative effect on pancreatic beta-cells, IL-22 constitutes an important factor potentially mediating post-surgery anti-diabetic and beneficial metabolic effects. It is noteworthy that the GK rat represents a lean diabetic model. Nevertheless, IL-22 is highly elevated post-surgery in these rats, indicating that its physiological roles are independent of weight.

In line with previous reports, acute or chronic treatment islets isolated from healthy human donors with IL-22 failed to enhance insulin secretion, suggesting that IL-22 is not a potentiator of insulin secretion per se, but that it might act to preserve or restore islet function under stressful conditions. Yet it remains unknown if IL-22 has a direct effect on compromised islets from diabetic donors. Among its multiple positive influences on metabolic disease, IL-22 injection in obese mice leads to increased serum PYY levels which is thought to contribute to the reduction in food intake and weight [21]. This increase in PYY is likely to be caused by IL-22-mediated stimulation of PYY expression in enteroendocrine L-cells, as indicated by our data. We only detected a mild increase in islet-derived PYY, which on its own, may be too small to directly impact on islet hormone secretion. Thus, it is likely that under physiological conditions, IL-22 indirectly modulates insulin secretion mainly via elevation in PYY release from L-cells (Fig. 6). Taken together our data demonstrate that PYY is a critical humoral factor which mediates enhanced islet secretory function, thus contributing to diabetes remission in humans upon bariatric surgery. Whereas there is no way to differentiate between the contribution of circulating and islet-derived PYY, it is likely they both contribute to restore islet function and sustain this effect over long-term. We postulate that modulation of blood-borne factors such as propionate, bile acids and IL-22 can increase overall PYY levels, which may subsequently influence islet function, resulting in improved insulin and glucagon secretion. Due to shortage of human serum samples we did not analyse changes in PYY [[3], [4], [5], [6], [7], [8], [9], [10], [11], [12], [13], [14], [15], [16], [17], [18], [19], [20], [21], [22], [23], [24], [25], [26], [27], [28], [29], [30], [31], [32], [33], [34], [35], [36]], which constitutes another major endogenous form of PYY likely to affect insulin release. Nevertheless, these findings provide new mechanistic links with key factors induced by bariatric surgery, restoration of impaired islet function and diabetes correction.

Bariatric surgery as a treatment option for type 2 diabetes (rather than for weight-loss alone) has been endorsed by 50 international health organizations. However, surgery remains invasive, irreversible and not suitable for all patients with T2D, highlighting the urgent need for medical therapies as alternatives. Using a unique translational research paradigm combining serum from patients before and following surgery with isolated human islets, our study demonstrates that the metabolic benefits of bariatric surgery can be emulated ex vivo, giving rise to the possibility of identifying the critical mediators of these effects. While our analysis focuses on the regulation of PYY and its effects on islet secretory functions, it is likely that other factors also contribute to achieve the metabolic benefits elicited by surgery, including improvements in peripheral tissue insulin sensitivity or tissue glucose clearance, and a combination of therapies may be required to successfully overcome the need for this invasive and irreversible procedure. Particularly, we foresee that the addition of PYY analogues to other incretin mimetics would potentiate and sustain their long-term efficacy. Moreover, the identification of factors specifically triggering PYY induction both systemically and within the islets, may pave the way for an alternative cure to peptide therapeutics, thus overcoming their inherent weaknesses, like poor pharmacokinetic properties and stabilities, and yet achieving the same benefits for the treatment of diabetes.

The following are the supplementary data related to this article.

Fig. S1 GLP-1 and insulin concentrations in serum samples before and after bariatric surgery. Total serum GLP-1(a) and insulin (b) in healthy volunteers (n = 20) and in patients before and 6 months after bariatric surgery (n = 25). Data are presented as mean ± SEM. (One-way ANOVA for multiple comparison) ^⁎^P < 0.05 for indicated comparison. Fig. S1

Fig. S2 Propionate and bile acids potentiate GSIS in mouse and human islets. (a) Insulin secretion was measured in mouse islets (mice n = 3) treated for 72 h with 1 mM propionate and then stimulated for 1 h with 1 mM (black bars) or 20 mM glucose (grey bars). (b) Insulin secretion was measured in human islets (donors n = 3) stimulated for 1 h with 1 mM (black bars) or 20 mM glucose (grey bars) in absence or presence of 30 μM GPBAR-A. Data are presented as percentage of basal secretion (mean ± SEM as percentage of content). ^⁎^P < 0.05, for indicated comparison. Fig. S2

Fig. S3 IL-22 is elevated in rats after gastric bypass and correlates with PYY increase in humans. (a) Plasma IL-22 levels in non-operated (n = 6), sham (n = 7) and RYGB (n = 21) GK rats. Data are presented as mean ± SEM. (One-way ANOVA for multiple comparison) ^⁎⁎⁎^P < 0.001 for indicated comparison. (b) Correlation between the increase in IL-22 and PYY in serum of obese patients before and six months after bariatric surgery (Pearson r: 0.73). Fig. S3

Fig. S4 Exposure of healthy islets with IL-22 does not potentiate GSIS. Insulin secretion in human (donors n = 3) (a, c) and mouse islets (b, d) was measured in the presence of 100 ng/mL IL-22 for 1 h (a, b) or 72 h (c, d) and stimulated with 1 mM (black bars) or 20 mM glucose (grey bars). Data are presented as percentage of basal secretion (mean ± SEM as percentage of content). Fig. S4

Supplementary material Image 1
