# Supplementary figures and images for: PYY plays a key role in the resolution of diabetes following bariatric surgery in humans
Source: eBioMedicine. 2019 Jan 11;40:67–76. doi: 10.1016/j.ebiom.2018.12.040 (PMC6413583; doi:10.1016/j.ebiom.2018.12.040)

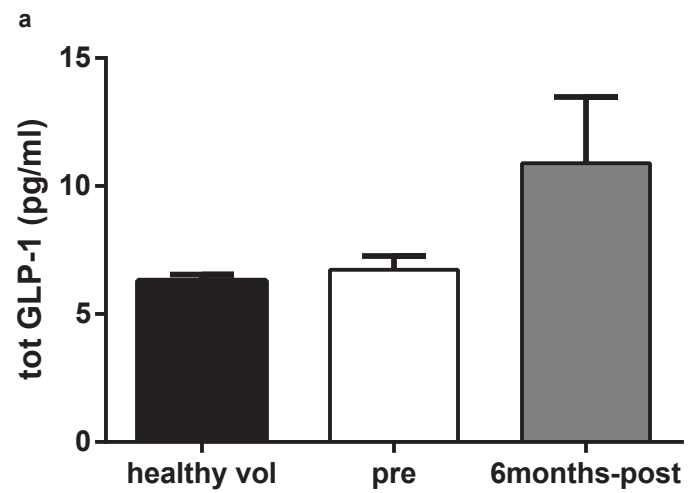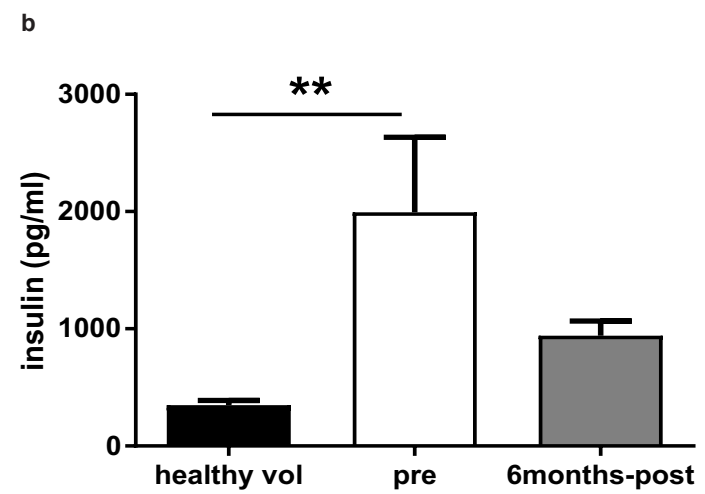

Supplement: Fig. S1 — GLP-1 and insulin concentrations in serum samples before and after bariatric surgery. Total serum GLP-1(a) and insulin (b) in healthy volunteers (n = 20) and in patients before and 6 months after bariatric surgery (n = 25). Data are presented as mean ± SEM. (One-way ANOVA for multiple comparison) ⁎P < 0.05 for indicated comparison. [file mmc1.pdf]

a

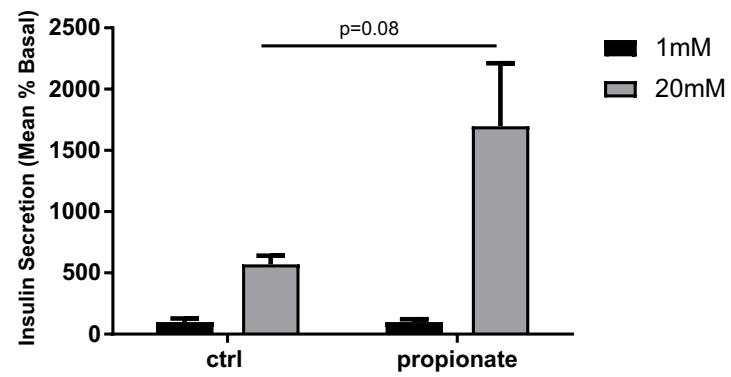

b

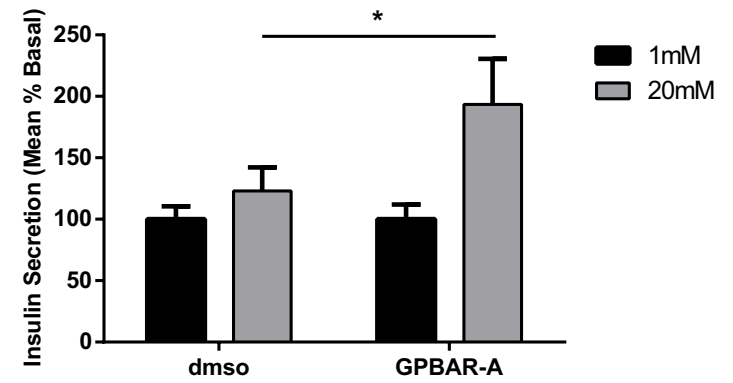

Supplement: Fig. S2 — Propionate and bile acids potentiate GSIS in mouse and human islets. (a) Insulin secretion was measured in mouse islets (mice n = 3) treated for 72 h with 1 mM propionate and then stimulated for 1 h with 1 mM (black bars) or 20 mM glucose (grey bars). (b) Insulin secretion was measured in human islets (donors n = 3) stimulated for 1 h with 1 mM (black bars) or 20 mM glucose (grey bars) in absence or presence of 30 μM GPBAR-A. Data are presented as percentage of basal secretion (mean ± SEM as percentage of content). ⁎P < 0.05, for indicated comparison. [file mmc2.pdf]

a

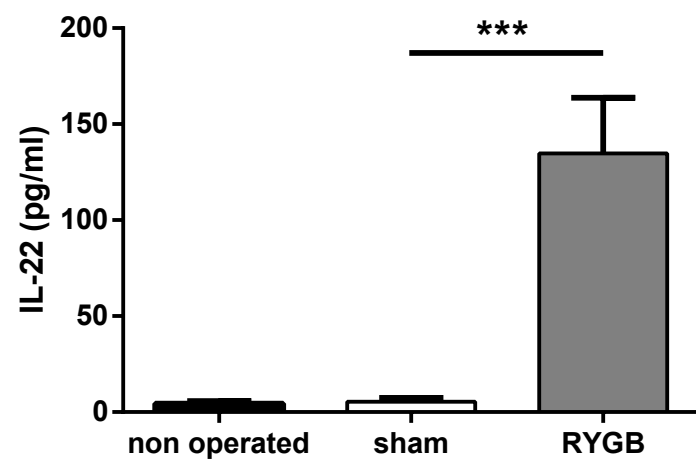

b

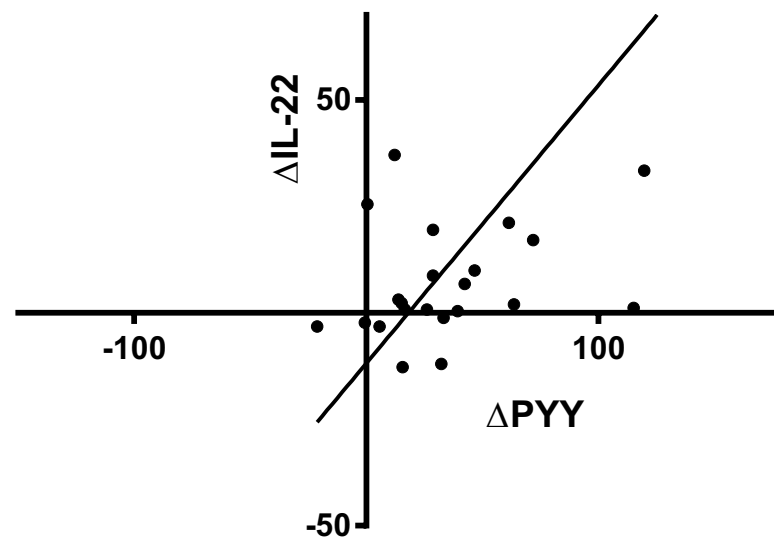

Supplement: Fig. S3 — IL-22 is elevated in rats after gastric bypass and correlates with PYY increase in humans. (a) Plasma IL-22 levels in non-operated (n = 6), sham (n = 7) and RYGB (n = 21) GK rats. Data are presented as mean ± SEM. (One-way ANOVA for multiple comparison) ⁎⁎⁎P < 0.001 for indicated comparison. (b) Correlation between the increase in IL-22 and PYY in serum of obese patients before and six months after bariatric surgery (Pearson r: 0.73). [file mmc3.pdf]

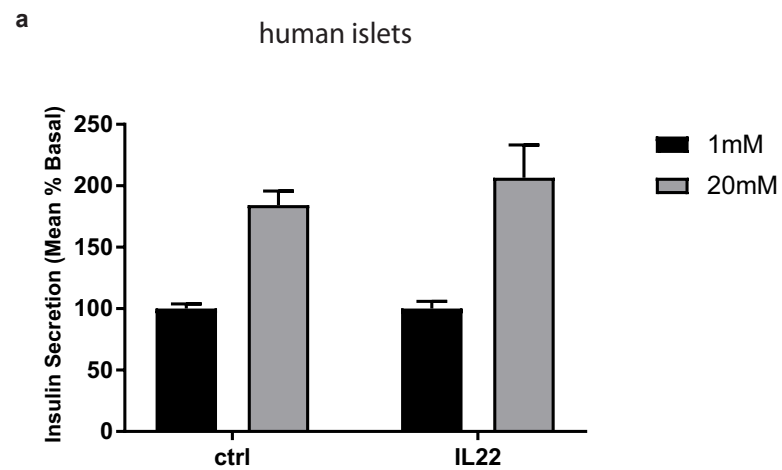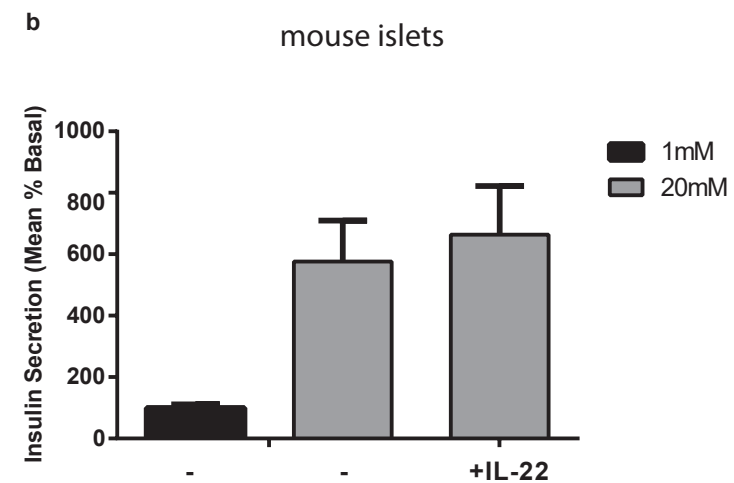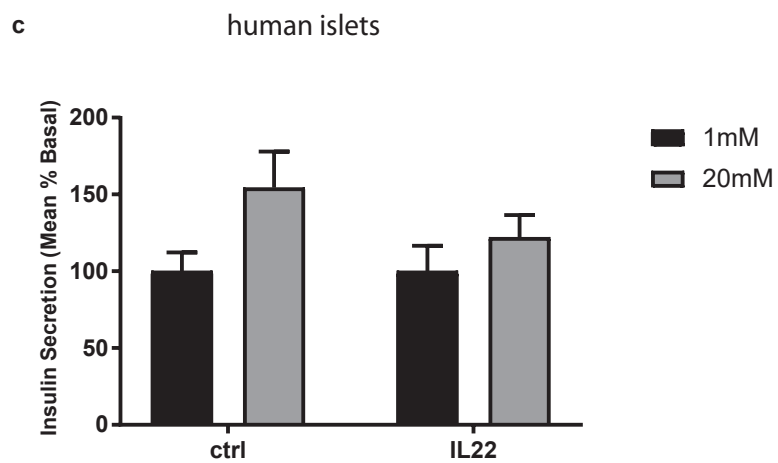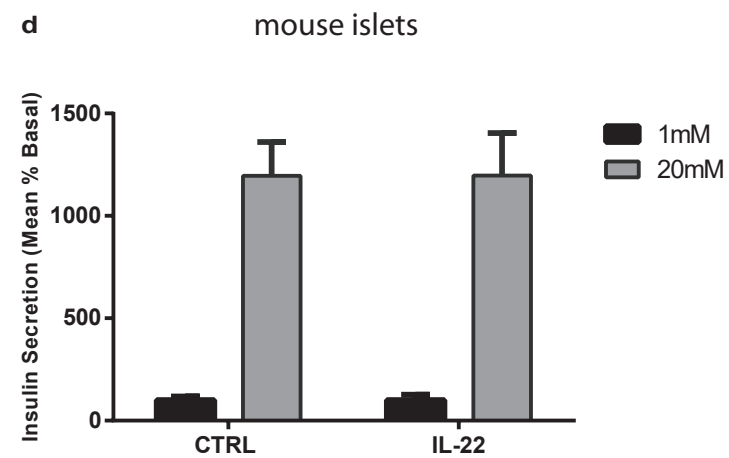

Supplement: Fig. S4 — Exposure of healthy islets with IL-22 does not potentiate GSIS. Insulin secretion in human (donors n = 3) (a, c) and mouse islets (b, d) was measured in the presence of 100 ng/mL IL-22 for 1 h (a, b) or 72 h (c, d) and stimulated with 1 mM (black bars) or 20 mM glucose (grey bars). Data are presented as percentage of basal secretion (mean ± SEM as percentage of content). [file mmc4.pdf]
