# Supplementary material for: PYY plays a key role in the resolution of diabetes following bariatric surgery in humans
Source: eBioMedicine. 2019 Jan 11;40:67–76. doi: 10.1016/j.ebiom.2018.12.040 (PMC6413583; doi:10.1016/j.ebiom.2018.12.040)
Supplement: Supplementary file 5 — Supplementary material [file mmc5.docx]

**Table S1.1 Patients details**

| **Table S1.1 Patient characteristics^*^**  (n=35) | |
| --- | --- |
| Age (yrs) | 49 (11) |
| Female (n, %) | 24 (67%) |
| Surgery (n, %)   - RYGB - SG | - 17 (49%) - 18 (51%) |
| BMI-pre (kg/m^2^) | 47 (8.4) |
| BMI-post (kg/m^2^) | 37.5 (7.5) |
| HbA1c pre (mmol/mol) | 40 (8.1) |
| HbA1c post (mmol/mol) | 34 (5.5) |
| ^*^mean +/- standard deviation unless specified | |

**Table S1.2 Healthy controls details**

| **Table S1.2 Healthy controls characteristics^*^**  (n=20) | |
| --- | --- |
| Age (yrs) | 48 (13.8) |
| Female (n, %) | 14 (70%) |
| BMI (kg/m^2^) | 22.2 (2.1) |
| ^*^mean +/- standard deviation unless specified | |

**Table S2. Human islet donor details**

| ID | Gender | Age (years) | BMI (kg/m2) | Diabetic status |
| --- | --- | --- | --- | --- |
| HP1631 | F | 25 | 23 | non |
| HP1659 | M | 46 | 27 | non |
| HP1734 | F | 46 | 35 | non |
| HP1738 | M | 49 | 31 | non |
| HP1741 | M | 46 | 28 | non |
| HP1742 |  | 30 | 25 | non |
| HP1744 | F | 57 | 24.2 | non |
| HP1745 |  | 57 | 32 | diabetic |
| HP1754 | M | 57 | 25.7 | non |
| HP1759 |  | 41 | 25 | non |
| HP1765 | M | 28 | 27 | non |
| P473 | M | 49 | 29 | non |
| P464 | M | 58 | 24 | non |
